# Exploring the Immunohistochemical Expression of Iron-Related Proteins in Non-Metastatic and Metastatic Feline Mammary Carcinomas

**DOI:** 10.3390/vetsci13080810

**Published:** 2026-08-15

**Authors:** Rebecca Leandri, Giorgia Rosato, Teresa Chianese, Evaristo Di Napoli, Manuela Martano, Karen Power

**Affiliations:** 1 Department of Biology, University of Naples Federico II, 80126 Naples, Italy; rebecca.leandri@unina.it (R.L.); giorgia.rosato@unina.it (G.R.); karen.power@unina.it (K.P.); 2 Department of Veterinary Medicine and Animal Productions, University of Naples Federico II, 80126 Naples, Italy; evaristo.dinapoli@unina.it (E.D.N.); manuela.martano@unina.it (M.M.)

**Keywords:** hypoxia, immunohistochemistry, iron metabolism, lymph node metastasis, Western blotting

## Abstract

Feline mammary tumors are the third most common neoplasms in female cats and they are characterized by high aggressiveness and high metastatic rates. Given the pivotal role of iron in human breast cancer development, in this study, we preliminarily explore the immunohistochemical expression of proteins involved in iron uptake, storage and efflux [Transferrin Receptor 1 (TfR1), Transferrin Receptor 2 (TfR2), ferritin (FTH1), and ferroportin (SLC40A1)] in non-metastatic and metastatic feline mammary carcinomas and their tributary lymph node and the possible correlation with hypoxia-inducible factor-1 (HIF-1). Our results showed an increased expression of TfR1 and TfR2 in relation to tumor progression. FTH1 immunolabeling was mainly observed in cancer cells delimiting necrotic areas and in lymph node metastasis, indicating greater iron storage possibly associated with hypoxic environments, as suggested by increased expression of HIF-1α. Also, an increase in SLC40A1 labeling in tumoral cells suggested greater iron efflux. Although preliminary, our results underline interesting differences between feline normal and tumoral mammary tissues, which could pave the way to further in vitro studies to better understand the role of iron in the progression of feline mammary tumors.

## Simple Summary

Feline mammary tumors are frequent tumors in female cats and are characterized by high aggressiveness and poor prognosis. Given the pivotal role of iron in human breast cancer development, in this study we preliminarily explore the immunohistochemical expression of proteins involved in iron uptake, storage and efflux, namely Transferrin Receptor 1 (TfR1), Transferrin Receptor 2 (TfR2), ferritin (FTH1), and ferroportin (SLC40A1) in non-metastatic and metastatic feline mammary carcinomas and their tributary lymph node. Our results showed an increased expression of uptake proteins in relation to tumor progression, with TfR2 labeling detected mainly in tumoral samples. FTH1 immunolabeling was observed in cancer cells delimiting necrotic areas and in lymph node metastasis, indicating greater iron storage possibly associated with hypoxic environments, as suggested by increased expression of the hypoxic-inducible factor 1α. An increase in SLC40A1 labeling in tumoral cells suggested greater iron efflux. Although preliminary, our results underline interesting differences between feline normal and tumoral mammary tissues, which could pave the way to further in vitro studies to better understand the role of iron in the progression of feline mammary tumors.

## 1. Introduction

Mammary tumors are the third most common neoplasm in female cats [1], mainly affecting pure-bred middle-aged to older non-neutered females [2,3]. An amount of 80–96% of feline mammary tumors are malignant [4,5,6,7], showing high aggressiveness, ulceration and invasion of lymphatic vessels with distant metastasis localized mainly in the lungs, pleura, liver and lymph nodes [6,8]. High metastatic rates and late diagnosis determine the poor prognosis of the disease even after surgical resection [9]. Mean overall survival time after diagnosis is 8–12 months [10,11,12]; however, survival time is influenced by different parameters such as tumor size (234 days) and lymph node invasion (232 days), which worsen overall survival [6,13]. Surgical resection is the therapeutic method of choice in managing feline mammary cancer (FMC), with chain mastectomy reducing local tumor recurrence compared to conservative surgery; however, survival rates and time remain low [14,15,16].

Feline mammary cancers have been proposed as effective translational models for studying tumorigenesis and for evaluating the therapeutic efficiency of new anti-neoplastic molecules, overcoming the limits of rodent models and accelerating translation of results for human and household pets [17,18]. Specifically, FMCs share similar epidemiological features, like age incidence and hormonal dependency, as well as key risk factors and genes involved in the onset and development of mammary gland cancer in humans [19,20]. Moreover, research suggests a similar mechanism of tumorigenesis based on the overexpression and amplification of cyclin A [21] and overexpression of p53 [22]. Interestingly, De Maria et al. (2005) [23] demonstrated great similarities between FMC and the HER2-type breast cancer in humans, both exhibiting poor responses to standard therapies.

In the quest for new therapeutic approaches, the role of iron and its metabolism has sparked the interest of researchers due to its role in cancer onset and progression [24]. Iron is an essential metal and cofactor of numerous enzymes and proteins involved in basic biological mechanisms like DNA synthesis, cell division, electron transport and respiration [25,26,27], and it has been reported that tumor cells can modify their iron metabolism pathways, increasing the cytoplasmic labile iron pool (cLIP) to satisfy their increased metabolic demands [28,29,30].

Recent studies in women have described the phenomenon of iron addiction and the role of iron-related proteins in the onset, growth and progression of breast cancer. The overexpression of iron uptake proteins (Transferrin Receptor 1 (TfR1) and Transferrin Receptor 2 (TfR2)) in breast cancer has been previously associated with cell proliferation and progression of the disease, as they increase the cLIP and sustain DNA synthesis and cell division [31,32,33]. Similarly, downregulation of the iron-exporter protein ferroportin, also known as solute carrier family 40 member 1 (SLC40A1), and consequent increased cLIP have also been associated with increased malignancy of breast cancer cells [34]. Moreover, the altered expression of ferritin, particularly of ferritin heavy chain (FTH1), has been linked to further alteration in intracellular iron availability and the progression and aggressiveness of breast cancer [35]. The relevance of iron homeostasis in breast cancer is not only connected to its role in cell proliferation and in Reactive Oxygen Species (ROS) production [36], but it is further highlighted by the interplay of iron and estrogen signaling. Estrogen metabolites can promote the reduction in ferritin-bound Fe^3+^ to Fe^2+^, enhancing the cLIP and ROS generation, thereby accelerating carcinogenesis [37,38,39]. Moreover, recent evidence has shown that hypoxic environments occurring in tumors can regulate iron-related proteins via the hypoxia-inducible factor-1 (HIF-1), increasing iron uptake and challenging iron homeostasis [40,41,42].

In veterinary medicine, despite the growing literature describing the alterations of iron metabolism in tumors and linking iron and cancer [43,44,45,46,47], the topic remains underexplored and largely uncharacterized.

Given their pivotal role in breast cancer, we here explore the expression of TfR1, TfR2, ferritin heavy chain (FTH1), and SLC40A1, investigating iron uptake, storage and efflux, and the possible relation with HIF-1α, in non-metastatic and metastatic FMCs and their lymph node metastasis. Results provide first insights into the possible role of iron-related proteins in FMC progression, paving the way to future more in-depth research.

## 2. Materials and Methods

### 2.1. Case Selection

Fifty-two samples were selected from the histological archives (years 2020–2025) of the Laboratory of Veterinary Pathology of the Department of Veterinary Medicine and Animal Productions of the University of Naples Federico II. Screening, case availability and selection were made using the informatics system MyClinical from the Laboratory of Veterinary Pathology. The histological reports were collected to obtain information about age, breed, histological diagnosis and the presence or absence of lymph node metastasis. Where metastasis was present in the draining lymph node, this was also analyzed. All information is reported in Table S1. Ethical committee approval and authorization for animal testing were not required, as all tissue specimens analyzed in this study were sourced from archived materials.

### 2.2. Histology

Collected samples had been fixed in 10% neutral buffered formalin before undergoing standard histopathological processing. Thin sections of 3 μm were obtained from paraffin-embedded tissue blocks and subsequently stained with hematoxylin and eosin (H&E) for microscopic analysis [48]. Histopathological classification of neoplastic lesions was performed based on the recently updated World Health Organization (WHO) classification [49]: healthy mammary gland tissue (HT), feline mammary carcinoma without lymph node metastasis (N0 FMCs), feline mammary carcinoma with lymph node metastasis (N+ FMCs), and the tributary lymph node that had the metastasis of the primary mammary carcinoma (LM). FMCs were further stratified by histological subtype and by grade.

### 2.3. Immunohistochemistry

Additional 3 μm sections were prepared for immunohistochemical (IHC) analysis to assess the expression of iron-related proteins TfR1 [50], TfR2, FTH1, SLC40A1 and HIF-1α following previously established protocols [45,51]. Feline liver tissues were used as positive controls, while negative controls were obtained by omitting primary Ab. Details regarding the antibodies used, including their dilutions, are provided in Table 1. Immunolabeling was visualized using diaminobenzidine tetrahydrochloride (DAB), followed by hematoxylin counterstaining. The specimens were examined and documented using a light microscope (AXIO SCOPE.A1, Carl Zeiss S.p.A., Oberkochen, Germany) equipped with a digital microphotography camera (Axiocam 105 color, Carl Zeiss S.p.A., Oberkochen, Germany) [52]. A semiquantitative immunostaining evaluation was performed by an experienced pathologist (M.M.) to ensure consistency following previously established protocols [50,53] and a range score (H-score) from 0 to 300 was obtained for each sample for each antibody. Using Prism 9.0.0. (GraphPad Software), statistical analysis was performed to verify differences among groups. Prior to one-way ANOVA, data were tested for normality using the Shapiro–Wilk test and for homogeneity of variances using Levene’s test. Since the assumptions were met, one-way ANOVA with Tukey’s multiple comparison test was used. A *p*-value of less than 0.05 was considered statistically significant.

### 2.4. Western Blotting

To evaluate the specificity of the immunohistochemical signals, Western blot analysis was conducted on frozen non-neoplastic feline mammary gland samples retrieved from the archives. Tissues were homogenized on ice using a Potter homogenizer in 5 mL of RIPA buffer composed of 50 mM Tris-HCl (pH 7.4), 150 mM NaCl, 1 mM EDTA, 10% NP-40, and a protease inhibitor cocktail (3 mM aprotinin, 1 mM leupeptin, 1 mM E-64, 130 μM bestatin, 1 mM EDTA, and 2 mM AEBSF) [54]. Homogenates were then centrifuged at 7000× *g* for 10 min at 4 °C. Protein concentration in the supernatants was quantified using a NanoDrop 1000 spectrophotometer (ThermoFisher Scientific, Carlsbad, CA, USA). Subsequently, 60 µg of total protein per sample was separated by 10% SDS–PAGE and transferred onto PVDF membranes (1620177, Bio-Rad, Hercules, CA, USA). Membranes were blocked for 1 h in TBS-T containing 5% non-fat dry milk (70166, Merck Millipore, Darmstadt, Germany), followed by overnight incubation at 4 °C with primary antibodies, as listed in Table 2. After washing in 0.1% TBS, membranes were incubated for 1 h at room temperature with horseradish peroxidase-conjugated secondary antibodies: anti-rabbit IgG (Santa Cruz, Cat. sc-2004) diluted 1:5000 in TBS-T with 2.5% BSA, and anti-mouse IgG (Santa Cruz, Cat. sc-2005) also diluted 1:5000 in TBS-T with 2.5% BSA.

Chemiluminescent detection was carried out using Clarity Western ECL substrate (1705061, Bio-Rad, Hercules, CA, USA) for 5 min at room temperature, and signals were visualized using the Chemidoc Touch Imaging System (Bio-Rad, Hercules, CA, USA). Each experiment was performed in biological triplicates to ensure reproducibility.

Since frozen neoplastic tissue samples were not available, antibody validation by Western blot was performed using non-neoplastic feline mammary tissues. Therefore, although the antibodies recognized proteins at the expected molecular weights, their performance in neoplastic tissues was not directly assessed.

## 3. Results

### 3.1. Histological Results

**Figure 1:**
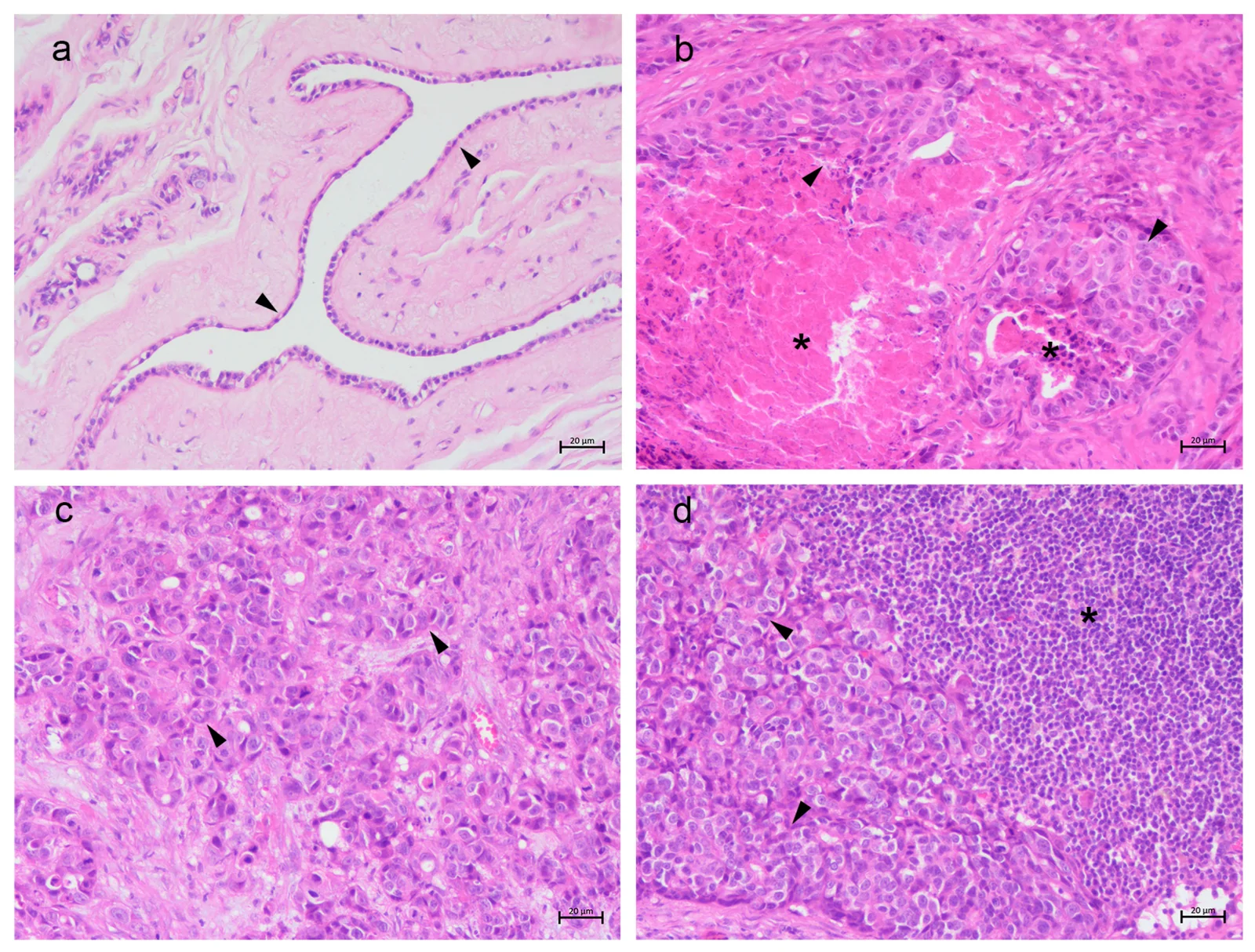
Feline mammary tissue. (**a**) Normal mammary gland with normal epithelial cells (arrow heads); (**b**) non-metastatic mammary carcinoma with tumoral cells (arrow heads) and necrosis (asterisks); (**c**) metastatic mammary carcinoma with tumoral cells (arrow heads); (**d**) tributary lymph node presenting lymphocytes (asterisk) and metastatic tumoral cells (arrow heads). Hematoxylin and eosin staining, 20×, scale bar 20 μm.

Histological evaluation of the FMC allowed diagnosis of seven normal mammary tissue samples (N1–N7; 7/52), fifteen feline mammary carcinomas without lymph node metastasis (N0 FMCs) (S1–S15; 15/52), fifteen feline mammary carcinomas with lymph node metastasis (N+ FMCs) (S16–S30; 15/52), and fifteen tributary lymph nodes that had the metastasis of the primary mammary carcinomas (LM) (S31–S45; 15/52) (Figure 1). Among the N0 and N+ groups, tubular carcinoma was the most frequent, followed by comedocarcinoma and solid carcinoma, whereas for both groups ductal and invasive micropapillary carcinoma occurred less frequently. No tubulopapillary carcinomas were observed in the N+ groups, while it was diagnosed in N0 samples. Detailed diagnosis can be found in Table S1.

### 3.2. Immunohistochemical Results

**Figure 2:**
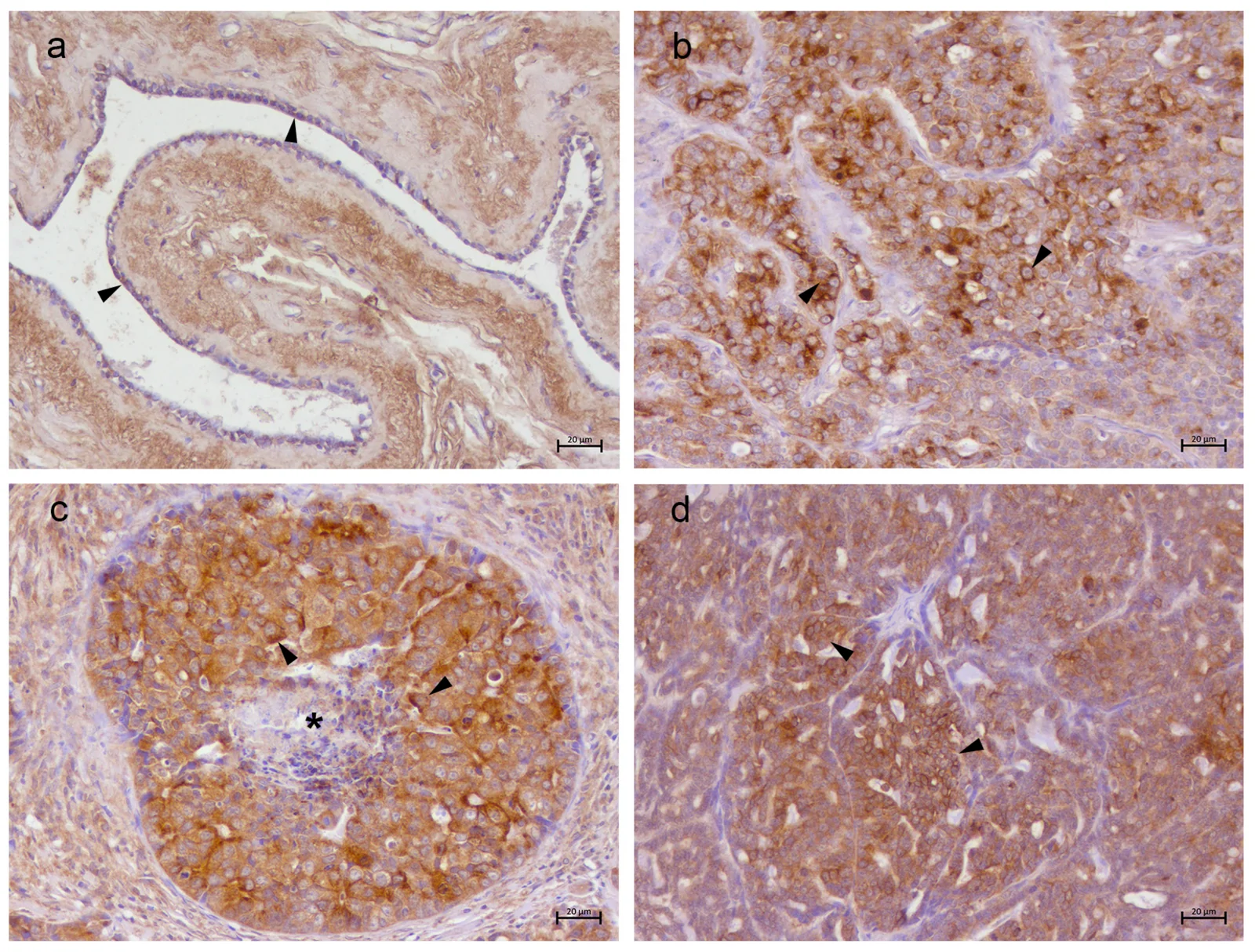
Feline mammary tissue. TfR1 immunostaining. (**a**) Normal mammary gland showing scattered membranous labeling in epithelial cells (arrow heads); (**b**) non-metastatic mammary carcinoma showing strong cytoplasmic labeling in tumoral cells (arrow heads); (**c**) metastatic mammary carcinoma showing strong cytoplasmic labeling in tumoral cells (arrow heads) and necrosis (asterisk); (**d**) tributary lymph node with metastatic cells showing cytoplasmic labeling (arrow heads). 20×, scale bar 20 μm.

**Figure 7:**
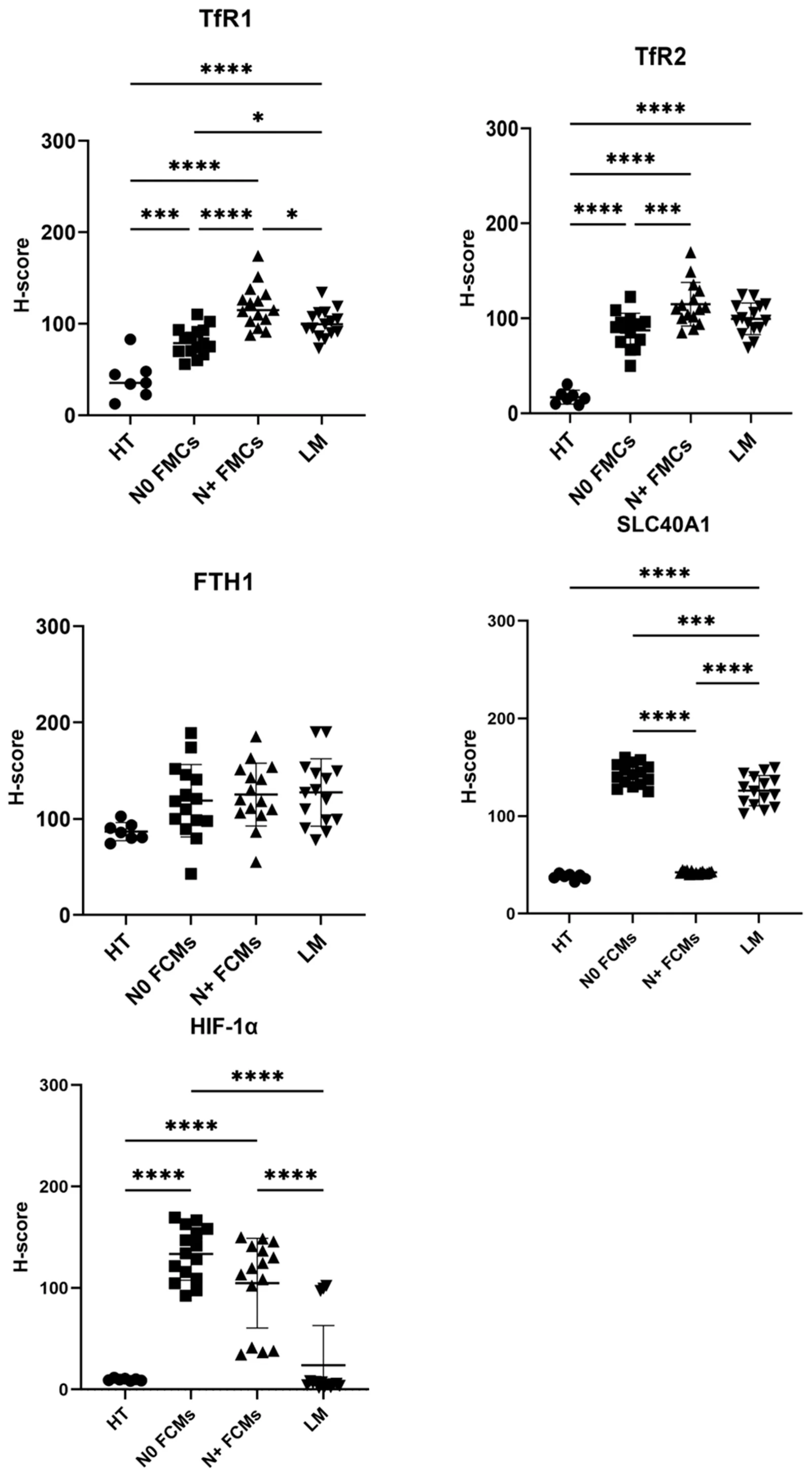
H-score of TfR1, TfR2, FTH1, SLC40A1 and HIF-1α expression in feline mammary healthy tissue (HT (n = 7)), feline mammary carcinoma without lymph node metastasis (N0 FMCs) (n = 15), feline mammary carcinoma with lymph node metastasis (N+ FMCs) (n = 15) and lymph node metastasis (LM) (n = 15). Data are presented as mean ± SD. **TfR1**: HT vs. N0 FMCs, *p* = 0.0001; HT vs. N+ FMCs, *p* < 0.0001; HT vs. LM, *p* < 0.0001; N0 FMCs vs. N+ FMCs, *p* < 0.0001; N0 FMCs vs. LM, *p* = 0.0315; N+ FMCs vs. LM, *p* = 0.0436; **TfR2**: HT vs. N0 FMCs, *p* < 0.0001; HT vs. N+ FMCs, *p* < 0.0001; HT vs. LM, *p* < 0.0001; N0 FMCs vs. N+ FMCs, *p* = 0.0009; N0 FMCs vs. LM, *p* = 0.2860; N+ FMCs vs. LM, *p* = 0.1114; **FTH1**: HT vs. N0 FMCs, *p* = 0.1591; HT vs. N+ FMCs, *p* = 0.0653; HT vs. LM, *p* = 0.0471; N0 FMCs vs. N+ FMCs, *p* = 0.9524; N0 FMCs vs. LM, *p* = 0.8962; N+ FMCs vs. LM, *p* = 0.9981; **SLC40A1**: HT vs. N0 FMCs, *p* < 0.0001; HT vs. N+ FMCs, *p* = 0.7752; HT vs. LM, *p* < 0.0001; N0 FMCs vs. N+ FMCs, *p* < 0.0001; N0 FMCs vs. LM, *p* = 0.0004; N+ FMCs vs. LM, *p* < 0.0001; **HIF-1α**: HT vs. N0 FMCs, *p* < 0.0001; HT vs. N+ FMCs, *p* < 0.0001; HT vs. LM, *p* = 0.8132; N0 FMCs vs. N+ FMCs, *p* = 0.1219; N0 FMCs vs. LM, *p* < 0.0001; N+ FMCs vs. LM, *p* < 0.0001. (* *p* < 0.1), (** *p* < 0.01), (*** *p* < 0.001), (**** *p* < 0.0001).

Immunohistochemistry of normal mammary gland tissue revealed membrane and cytoplasmic TfR1 immunolabeling in all mammary gland tissues (Figure 2a–d; Figure S6). The H-score showed an increased protein level of TfR1 in N0 FMCs (80.70 ± 14.90) versus healthy mammary tissue (mean 40.02 ± 20.86) (*p* < 0.01). Moreover, a marked upregulation of TfR1 expression was also observed in both the N+ FMCs (mean 119.34 ± 22.56) and LM (mean 100.47 ± 14.86) groups compared to the HT baseline (*p* < 0.0001 for both comparisons) and to the N0 FMCs group (Figure 7). These findings suggest a strong association between TfR1 overexpression and the progression of the tumor.

**Figure 3:**
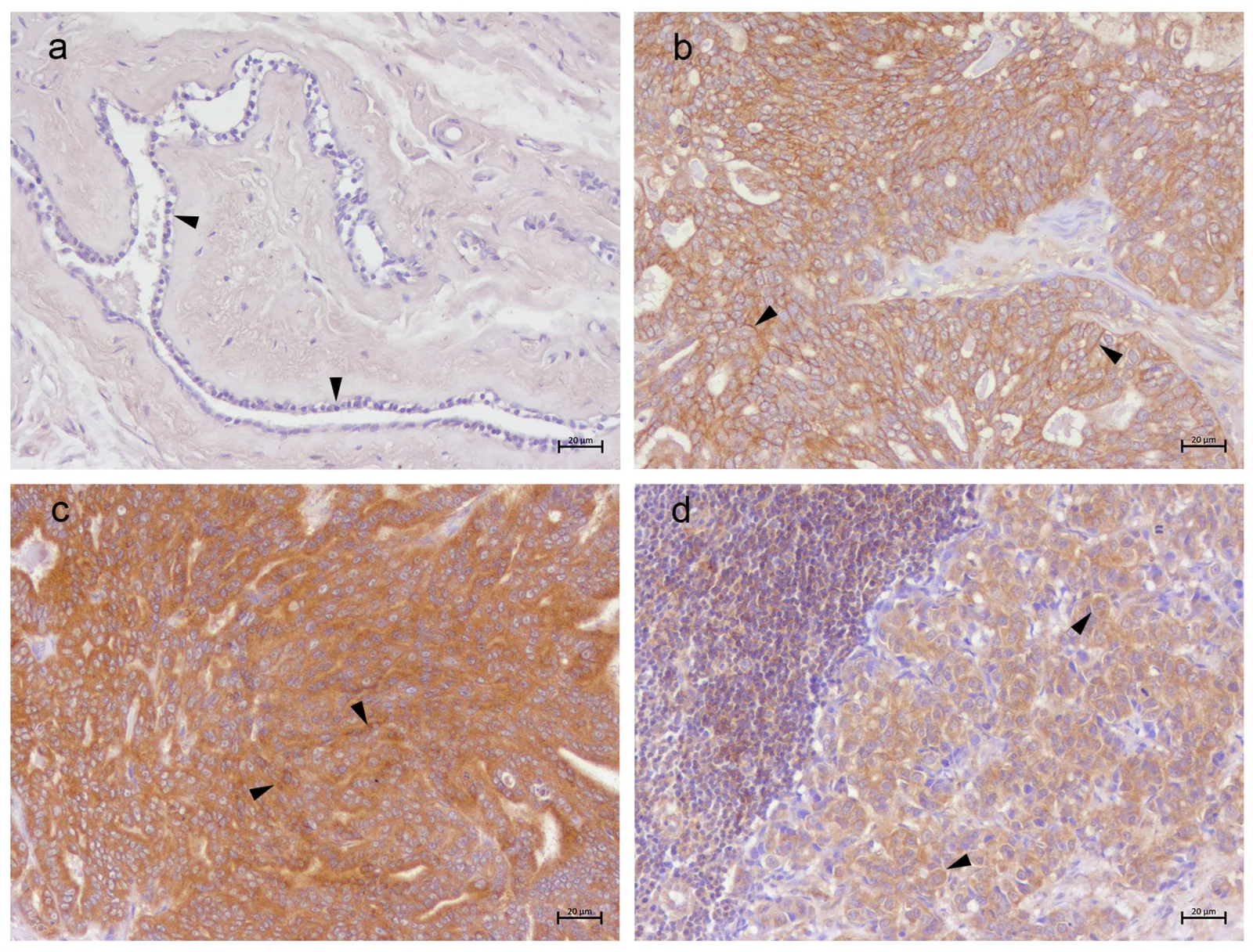
Feline mammary tissue. TfR2 immunostaining. (**a**) Normal mammary gland showing few labeled epithelial cells (arrow heads); (**b**) non-metastatic mammary carcinoma showing strong membranal labeling in tumoral cells (arrow heads); (**c**) metastatic mammary carcinoma showing very strong cytoplasmic labeling in tumoral cells (arrow heads); (**d**) tributary lymph node with metastatic cells showing cytoplasmic labeling in tumoral cells (arrow heads), 20×, scale bar 20 μm.

TfR2 was poorly detected in normal tissues of the feline mammary gland (mean 16.94 ± 6.77), but TfR2 labeling was detected in all tumoral samples (Figure 3a–d; Figure S7) with an increase in H-score following tumoral progression. In particular, TfR2 expression was significantly increased in the N0 FMCs group (mean 87.30 ± 17.37) compared with the HT group (*p* < 0.0001) and further increased in the N+ FMCs (114.77 ± 22.24), which showed significantly higher H-scores than both HT (*p* < 0.0001) and N0 FMCs (*p* = 0.0009). The LM group (99.35 ± 16.04) also exhibited significantly higher TfR2 expression compared to HT (*p* < 0.0001), although no significant differences were detected between LM and either N0 or N+ FMCs (Figure 7).

**Figure 4:**
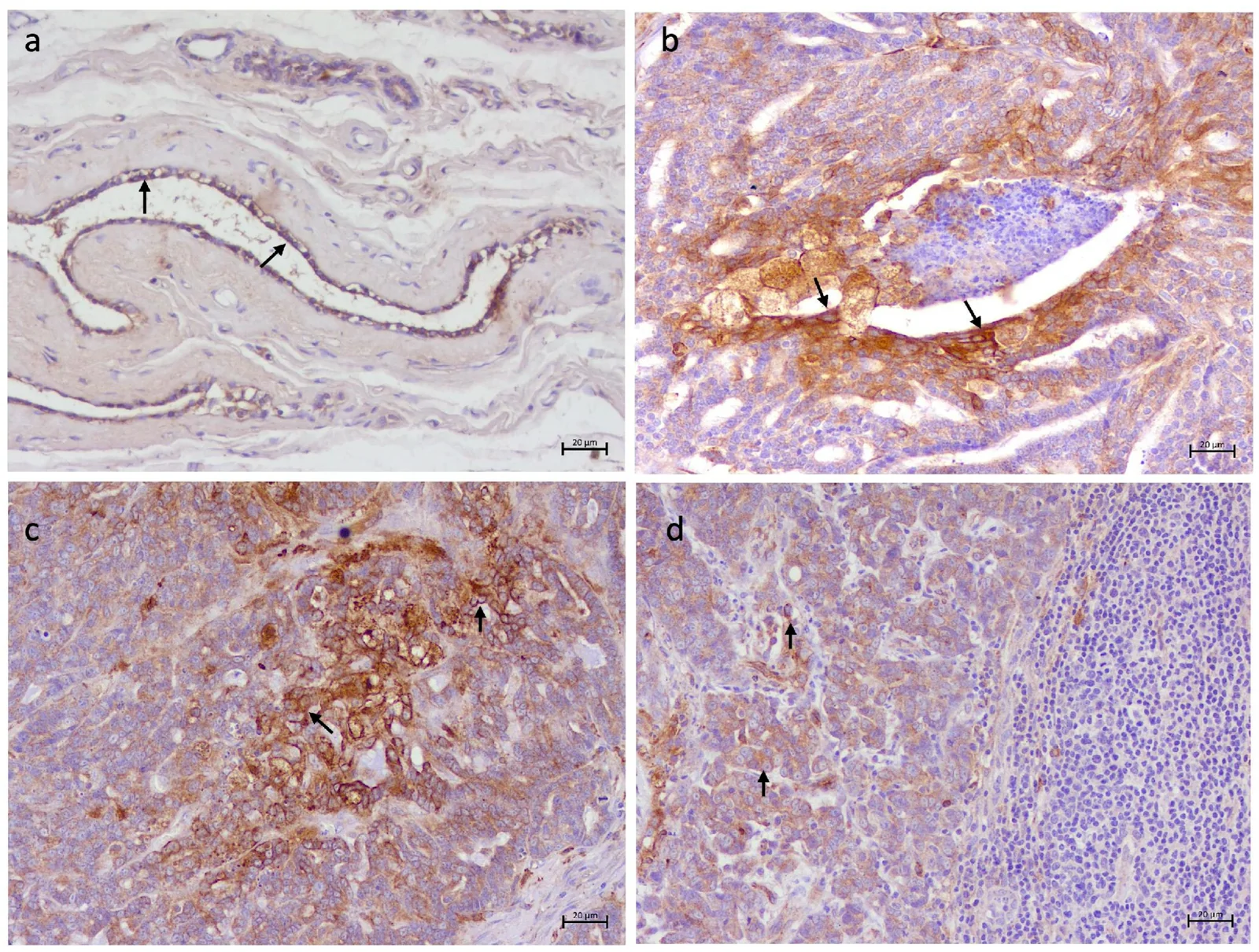
Feline mammary tissue. FTH1 immunostaining. (**a**) Normal mammary gland showing few labeled epithelial cells (arrow); (**b**) non-metastatic mammary carcinoma showing strong labeling in tumoral cells (arrow); (**c**) metastatic mammary carcinoma showing strong cytoplasmic labeling in tumoral cells (arrow); (**d**) tributary lymph node with metastatic cells showing cytoplasmic labeling in tumoral cells (arrow), 20×, scale bar 20 μm.

FTH1 was expressed in normal feline mammary gland tissues with a relatively moderate H-score (86.75 ± 8.80). In contrast, all feline mammary carcinoma groups showed increased FTH1 expression. Specifically, FTH1 immunoreactivity was significantly higher in the N0 FMCs (118.91 ± 36.32), N+ FMCs (125.26 ± 31.51), and LM (127.37 ± 33.79) groups compared with HT, whereas no significant differences were detected among the tumoral groups (Figure 7). Interestingly, FTH1 was highly detected in tumoral cells surrounding comedonic areas (Figure 4a–d; Figure S8).

**Figure 5:**
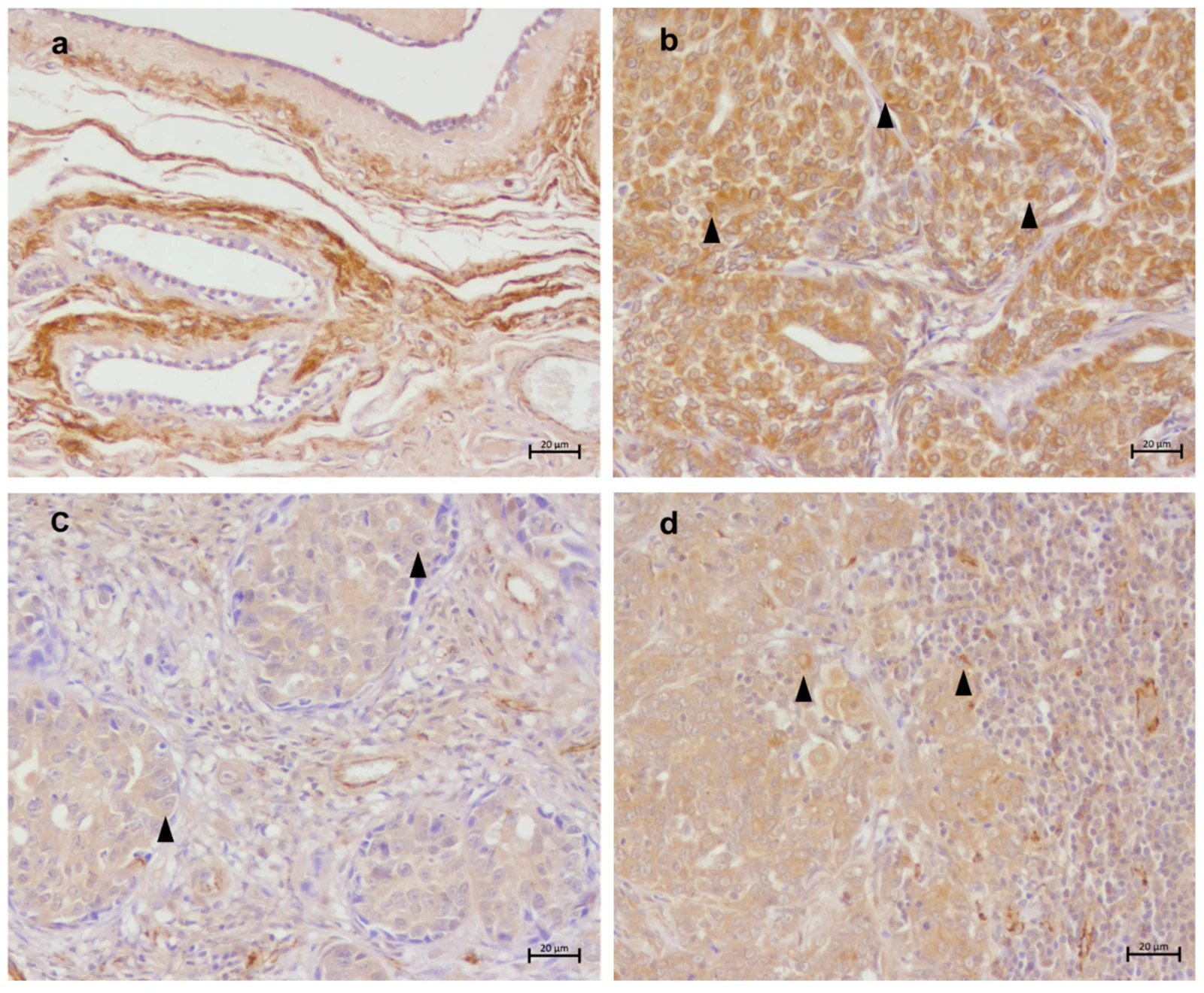
Feline mammary tissue. SLC40A1 immunostaining. (**a**) Normal mammary gland not showing labeled epithelial cells; (**b**) non-metastatic mammary carcinoma showing strong cytoplasmic labeling in tumoral cells (arrow heads); (**c**) metastatic mammary carcinoma showing weak cytoplasmic labeling in a few tumoral cells (arrow heads); (**d**) tributary lymph node with metastatic cells showing cytoplasmic labeling in tumoral cells (arrow heads), 20×, Scale bar 20 μm.

SLC40A1 immunoreactivity was low in both normal and N+FMCs with comparable H-scores (respectively 37.76 ± 3.08 and 42.30 ± 1.55). While, SLC40A1 expression increased markedly in N0 FMCs and in LM (Figure 5a–d; Figure S9). Specifically, both the N0 FMCs and LM groups showed significantly higher H-scores (respectively, 142.5 ± 11.18 and 125.95 ± 15.48) and immunolabeling than the N+ FMCs group (*p* < 0.0001 for both comparisons), whereas no significant differences were observed between LM and N0 FMCs (Figure 7).

**Figure 6:**
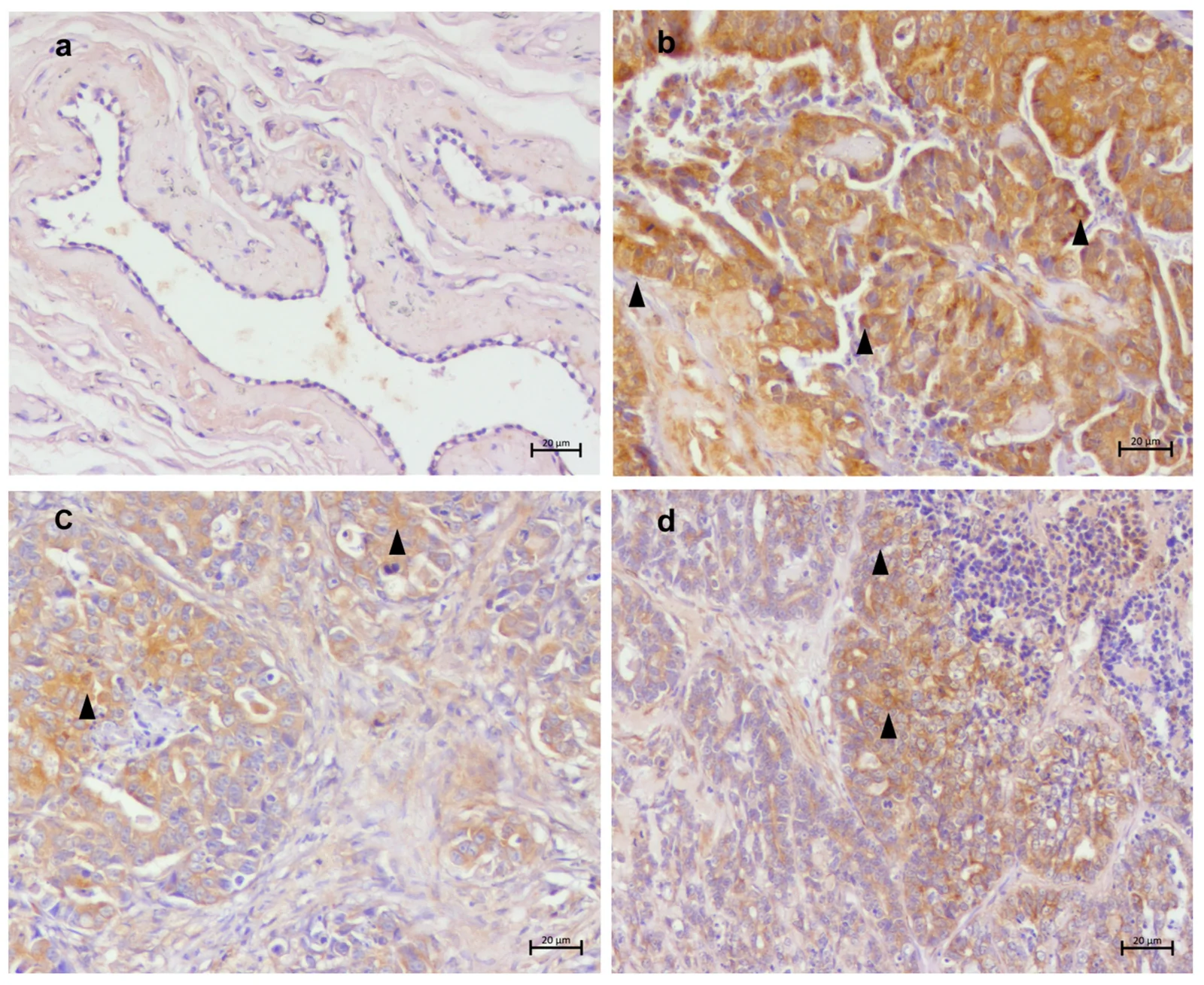
Feline mammary tissue. HIF-1α immunostaining. (**a**) Normal mammary gland not showing labeled cells; (**b**) non-metastatic mammary carcinoma showing strong cytoplasmic labeling in tumoral cells (arrow heads); (**c**) metastatic mammary carcinoma showing cytoplasmic labeling in a few tumoral cells (arrow heads); (**d**) tributary lymph node with metastatic cells showing moderate cytoplasmic labeling in tumoral cells (arrow heads), 20×, scale bar 20 μm.

HIF-1α was poorly detected in normal tissues of the feline mammary gland with the H-score of 9.01 ± 1.04. Whereas HIF-1α labeling was observed in all tumoral groups (Figure 6a–d; Figure S10), in particular it was higher in the N0 FMCs group (*p* < 0.0001) with a H-score of 133.62 ± 25.03. Moreover, the N+ FMCs group maintained high HIF-1α immunoreactivity, with a high H-score (104.86 ± 42.69). Conversely, the LM group exhibited a significant reduction in HIF-1α expression compared with the N0 FMCs group (*p* < 0.0001), with an H-score of 23.95 ± 37.87 (Figure 7).

### 3.3. Western Blotting Results

**Figure 8:**
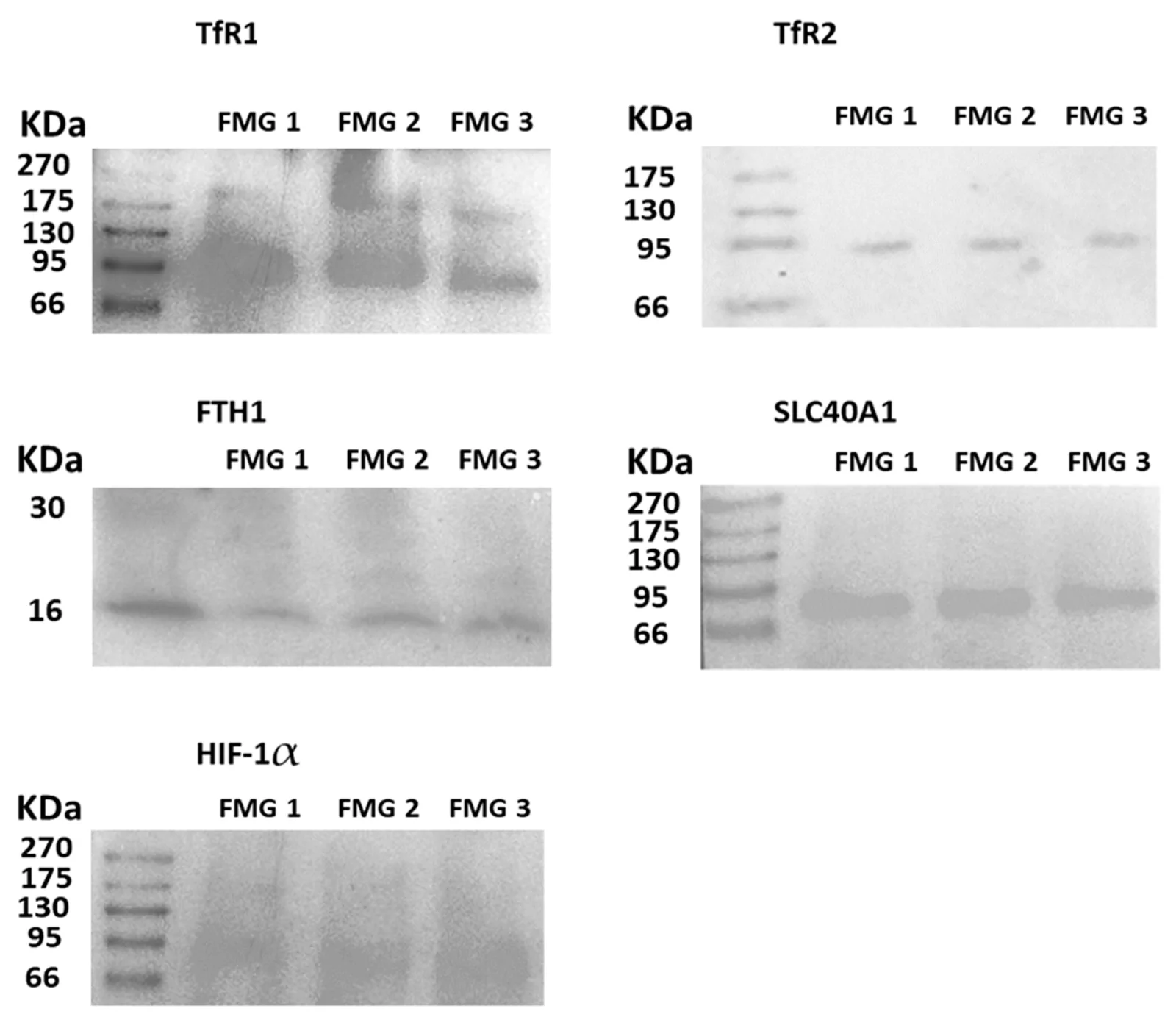
Representative TfR1 (~90 kDa), TfR2 (~100 kDa), FTH1 (~20 kDa), SLC40A1 (~62–70 kDa) and HIF-1α (~93 kDa) immunoblot analysis in 3 non-neoplastic feline mammary glands (FMGs) (See Figures S1–S5).

The specificity of the antibodies against TfR1, TfR2, FTH1, SLC40A1 and HIF-1α was confirmed via Western blot analysis on total protein lysates derived from non-neoplastic feline mammary gland tissues. As shown in Figure 8, the anti-TfR1 antibody detected an immunoreactive band at approximately 90 kDa while the anti-TfR2 antibody detected a single immunoreactive band at approximately 100 kDa. Also, an immunoreactive band was observed at 21 kDa. Following anti-FTH1F or SLC40A1, two immunoreactive bands were observed between approximately 62 and 70 kDa. Similar findings have been previously described for ferroportin and are thought to reflect different forms of the protein. Moreover, an immunoreactive band was also observed at 93 kDa following anti-HIF-1α binding. These bands correspond to the expected molecular weights of the respective feline protein isoforms, supporting the specificity of the antibodies.

## 4. Discussion

Iron is an essential trace element involved in fundamental biological processes, including DNA synthesis, cellular respiration, and cell proliferation [25,26,27]. However, the tight regulation of iron homeostasis is critical, especially in tissues with high proliferative rates, such as neoplastic ones [55]. Indeed, if, on one hand, iron sustains DNA synthesis and cell proliferation, on the other hand, its redox-active nature renders it potentially cytotoxic when present in excess, promoting oxidative stress through the Fenton reaction and leading to ferroptosis [56,57,58,59].

In breast cancer, upregulation of the expression of iron uptake proteins such as TfR1 and TfR2 was reported [60]. These findings overlap with those observed in our study, further supporting the possibility of using natural FMC as a model for human breast cancer also in the study of iron-related tumorigenesis and progression [61]. Our findings indicate that iron uptake is dynamically remodeled during FMC progression rather than being uniformly dysregulated. The expression patterns of the investigated proteins differed between non-metastatic primary tumors (N0 FMCs), metastatic primary tumors (N+ FMCs), and lymph node metastases (LM), suggesting that distinct mechanisms characterize different stages of disease progression. A previous study by Marques et al. (2017) [61] evaluated the expression of iron-related proteins in benign and malignant feline mammary tumors, but no significant differences were observed according to malignancy, while a study by Rensi et al. (2021) [50] described a correlation between tumor progression and expression of TfR1. Overexpression of TfR1 is a hallmark of several cancers, including mammary carcinoma, and it is associated with increased iron uptake, enhanced proliferation, and worse clinical outcomes [62,63,64,65,66,67,68], as iron overload can be associated with the progression of mammary cancer toward a more malignant phenotype [69]. Similarly, although less studied in veterinary medicine, TfR2 may contribute to tumorigenesis through iron-mediated mechanisms [51,70,71,72,73] and hormone signaling crosstalk, including estrogen responsiveness [74]. In human oncology, the role of TfR2 in Triple-Negative Breast Cancer (TNBC) was investigated through in vitro experiments [33]. The overexpression of TfR2 in TNBC cells compared with that of healthy breast epithelial cells was highlighted and its oncogenic effects were demonstrated. Moreover, it was described that TfR2 knockdown could inhibit TNBC cell proliferation by activating ferroptosis, an iron-dependent non-apoptotic cell death [75]. Although TfR2 has been found upregulated in several tumors, its connection to prognosis is still debated [76]. In our study, both TfR1 and TfR2 were poorly detected in normal mammary tissues, while they presented progressive expression according to malignancy, suggesting a possible use of iron uptake proteins as markers for FMCs progression.

On the one hand, in our study, we observed a possible increase in the uptake of iron; the expression of SL40A1 appears controversial, as it was less expressed in N+FMCs and more expressed in N0 FMCs and LM. SL40A1 is the only mechanism for exporting intracellular non-heme iron and has a central role in iron homeostasis and cLIP amounts. To date, SL40A1 has not been extensively studied in the context of cancer and its role in cancerogenesis is not fully understood. Nevertheless, it appears to be decreased in human breast cancer epithelial cells when compared to normal breast cells, with the lowest ferroportin expression associated with the highest severity and highest amounts of cLIP [34]. Consequently, our results appear slightly in contrast with those reported in breast cancer and the role of SL40A1 needs to be further elucidated. Nevertheless, the downregulation of the iron efflux mechanism in N+ FMCs could lead to increased amounts of cLIP, which in turn could be used to sustain tumor growth and cell metastasis, as previously described [77]. The upregulation of SL40A1 in N0 FMCs and LM is controversial and, currently, we can only hypothesize possible underlying mechanisms. On one hand, efflux increase could be read as a possible cell protective mechanism. Indeed, it has been demonstrated that increased iron efflux impeded tumor growth and metastasis, and inhibited epithelial–mesenchymal transition [77]. On the other hand, we cannot exclude that, by increasing iron efflux, tumoral cells are attempting to reduce the amount of cLIP and fight ferroptosis, which would benefit cancer cell survival. More likely, the differential expression of SL40A1 between N0 FMCs, N+FMCs and LM could be a consequence of other factors such as biological heterogeneity and microenvironmental differences, or even technical variation.

Ferritin, particularly ferritin heavy chain 1 (FTH1), can play a double role in cancer onset. On one hand, it sequesters excess intracellular iron, limiting oxidative damage [78]; on the other hand, high levels of ferritin are associated with immunosuppression and enhanced tumor cell survival [79,80]. In breast cancer, ferritin appears upregulated [60], and elevated ferritin levels in tissue have been correlated with tumor grade, epithelial proliferation, recurrence risk [35,81] and hypoxic conditions. The first results provided by our study show increased detection of ferritin in all tumoral samples. In N0 and N+ FMCs, FTH1 overexpression could be related to the hypoxic tumoral environment and the increased expression of HIF-1α. Indeed, iron metabolism is highly affected by hypoxia and the mechanisms of intake, storage, use and efflux appear modified. Particularly, in hypoxic contexts, TfR1 and FTH1 are usually up-regulated to sustain the higher iron requirements and to prevent ferroptosis through iron storage. On the contrary, as LM presented a very diverse expression of HIF-1α, increased iron storage cannot be justified solely by a hypoxic environment, but it is possibly due to a specific tumoral microenvironment different from that of primary tumors and resulting from the specific biological behavior of a particular cancer type. Particularly, the absence of necrosis in the analyzed LM samples might play a role in HIF-1α downregulation, given that HIF-1α is typically upregulated in necrotic areas. A greater number of samples and in vitro studies will help shed light on the uncertainties of the present study.

## 5. Conclusions

In many cancers, iron metabolism pathways are often altered, suggesting that reprogramming of iron metabolism is a key feature of tumorigenesis. The promotion of the activity of iron-related proteins in tumors can create a new balance able to feed the “iron addiction”, sustain progression and prevent damage caused by iron overload. Since the number of samples is limited, with a low number of healthy mammary tissues, the present findings should be interpreted with caution. Also, the limited sample size prevented any meaningful subtype-specific analyses and no correlation with clinicopathological data was therefore performed. Future studies should correlate the expression of iron-related proteins while also considering parameters like tumor size and grade and survival time. Nevertheless, our study confirms the alteration in the expression of iron-related proteins under the immunohistochemical profile in FMCs, providing a first glance at possible alterations in iron metabolism. If iron uptake appears to follow cancer progression also in FMCs, iron storage and efflux do not seem to follow this rule, possibly due to the different biological characteristics of different cancer types. Unfortunately, many of the proposed mechanisms underlying our results have not been fully investigated, due to the inherent constraints of a retrospective study that lacks standardized collection protocols; nevertheless, starting from this “static picture”, more dynamic in vitro approaches could better elucidate the hypothesis here provided.

## Figures and Tables

**Table 1:** Primary antibodies used for immunohistochemistry analysis.

Antibody	Manufacturer/Clone	Host Species	Dilution
TfR1	ThermoFisher 13-6800	Mouse	1:100
TfR2	Antibodies ABIN2782221/Polyclonal	Rabbit	1:100
FTH1	Antibodies ABIN2785803/Polyclonal	Rabbit	1:100
SLC40A1	Elabscience E-AB-19866/Polyclonal	Rabbit	1:100
HIF-1α	Abcam ab1/Polyclonal	Rabbit	1:100

**Table 2:** Details about primary antibodies used in Western blot analysis.

Antibody	Manufacturer/Clone	Host Species	Dilution
TfR1	ThermoFisher, 13.6800	Mouse	1:2000
TfR2	Antibodies ABIN2782221/Polyclonal	Rabbit	1:1000
FTH1	Antibodies ABIN2785803/Polyclonal	Rabbit	1:500
SLC40A1	Elabscience E-AB-19866/Polyclonal	Rabbit	1:1000
HIF-1α	Abcam ab1/Polyclonal	Rabbit	1:1000

## Data Availability

The original contributions presented in this study are included in the article/Supplementary Materials. Further inquiries can be directed to the corresponding author.

## Supplementary Materials

The following supporting information can be downloaded at https://www.mdpi.com/article/10.3390/vetsci13080810/s1, Table S1: breeds, age and histopathological diagnosis of 52 feline mammary tissue samples; Figures S1–S5: whole Western blots; Figures S6–S10: high-magnification of immunohistochemical images.
