# Supplementary material for: Exploring the Immunohistochemical Expression of Iron-Related Proteins in Non-Metastatic and Metastatic Feline Mammary Carcinomas
Source: Vet Sci. 2026 Aug 15;13(8):810. doi: 10.3390/vetsci13080810 (PMC13517384; doi:10.3390/vetsci13080810)
Supplement: Supplementary file 1 [file vetsci-13-00810-s001.zip › vetsci-4461234-supplementary.pdf]

Table S1. Breeds, age and histopathological diagnosis of 52 feline mammary tissue samples.

Samples	Breed	Age (ys)	Classification	Histopathological diagnosis
S1	European mixed breed	10	N0 FMCs	Comedocarcinoma grade II
S2	European mixed breed	8	N0 FMCs	Tubular carcinoma grade II
S3	European mixed breed	12	N0 FMCs	Comedocarcinoma grade III
S4	European mixed breed	11	N0 FMCs	Solid carcinoma grade III
S5	European mixed breed	10	N0 FMCs	Ductal carcinoma grade II
S6	European mixed breed	8	N0 FMCs	Tubular carcinoma grade II
S7	Chartreux	9	N0 FMCs	Tubular carcinoma grade III
S8	European mixed breed	11	N0 FMCs	Comedocarcinoma grade II
S9	European mixed breed	14	N0 FMCs	Solid carcinoma grade II
S10	European mixed breed	6	N0 FMCs	Comedocarcinoma grade III
S11	Persian	7	N0 FMCs	Tubular carcinoma grade I
S12	European mixed breed	8	N0 FMCs	Tubulopapillary carcinoma grade II
S13	European mixed breed	6	N0 FMCs	Tubular carcinoma grade III

S14	European mixed breed	10	N0 FMCs	Invasivemicropapillary carcinoma grade II
S15	European mixed breed	11	N0 FMCs	Tubular carcinoma grade II
S16	European mixed breed	9	N+ FMCs	Solid carcinoma grade III
S17	European mixed breed	9	N+ FMCs	Ductal carcinoma grade II
S18	Ragdoll	10	N+ FMCs	Tubular carcinoma grade II
S19	European mixed breed	11	N+ FMCs	Comedocarcinoma grade III
S20	Persian	13	N+ FMCs	Tubular carcinoma grade III
S21	European mixed breed	8	N+ FMCs	Comedocarcinoma grade II
S22	European mixed breed	10	N+ FMCs	Solid carcinoma grade III
S23	European mixed breed	11	N+ FMCs	Tubular carcinoma grade I
S24	Scottish fold	10	N+ FMCs	Comedocarcinoma grade II
S25	European mixed breed	7	N+ FMCs	Solid carcinoma grade II
S26	Chartreux	15	N+ FMCs	Tubulopapillary carcinoma grade II
S27	Persian	13	N+ FMCs	Invasive micropapillary carcinoma grade II
S28	European mixed breed	7	N+ FMCs	Comedocarcinoma grade III
S29	European mixed breed	9	N+ FMCs	Ductal carcinoma grade II

S30	European mixed breed	11	N+ FMCs	Comedocarcinoma III
S31	European mixed breed	9	LM	Solid carcinoma
S32	European mixed breed	9	LM	Ductal carcinoma
S33	Ragdoll	10	LM	Tubular carcinoma
S34	European mixed breed	11	LM	Comedocarcinoma
S35	Persian	13	LM	Tubular carcinoma
S36	European mixed breed	8	LM	Comedocarcinoma
S37	European mixed breed	10	LM	Solid carcinoma
S38	European mixed breed	11	LM	Tubular carcinoma
S39	Scottish fold	10	LM	Comedocarcinoma
S40	European mixed breed	7	LM	Solid carcinoma
S41	European mixed breed	15	LM	Tubulopapillary carcinoma
S42	European mixed breed	8	LM	Invasive micropapillary carcinoma
S43	European mixed breed	12	LM	Comedocarcinoma
S44	European mixed breed	7	LM	Ductal carcinoma
S45	European mixed breed	9	LM	Comedocarcinoma

N1	European mixed breed	13	HT
N2	European mixed breed	14	HT
N3	European mixed breed	14	HT
N4	European mixed breed	12	HT
N5	Persian	14	HT
N6	European mixed breed	11	HT
N7	European mixed breed	10	HT

N0 FMCs: Feline mammary carcinoma without lymph node metastasis; N+ FMCs: Feline mammary carcinoma with lymph node metastasis; LM: tributary lymph node with metastasis; HT: healthy mammary gland tissue

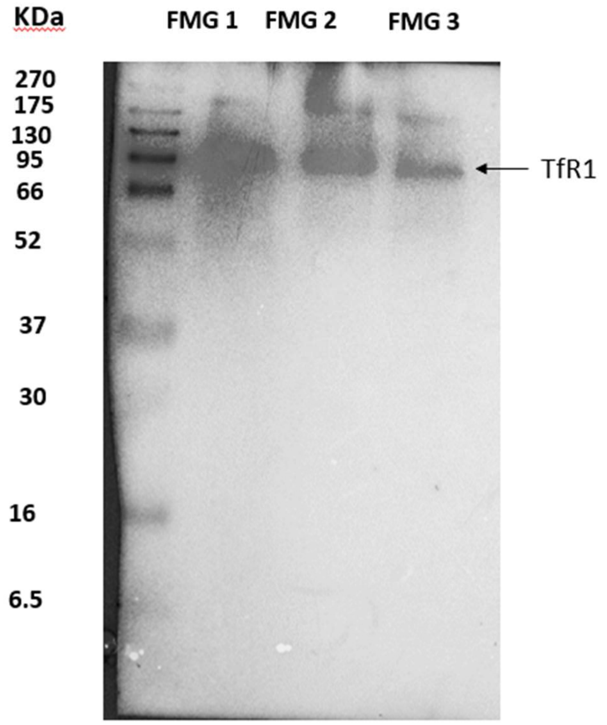

Figure S1. Western blot membrane of TfR1 (~90 kDa) protein detected with anti-Transferrin receptor 1 (Thermofisher H68.4, 1:2000; Carlsbad, USA) antibody.

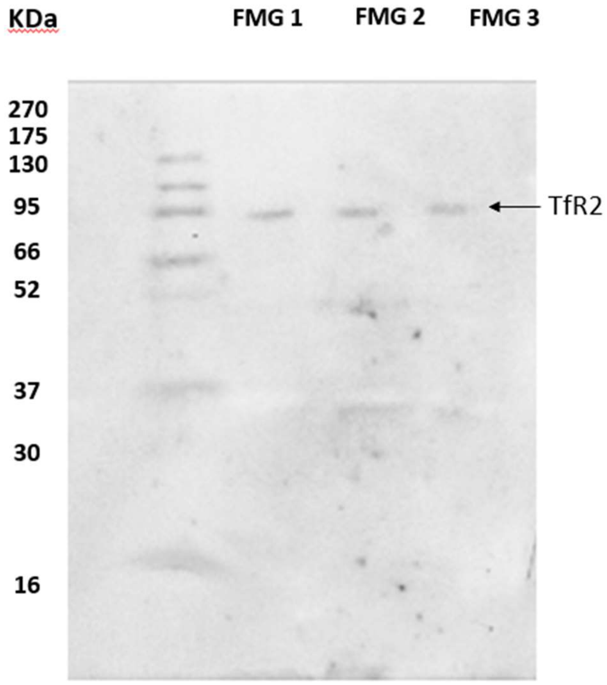

Figure S2. Western blot membrane of TfR2 (~100 kDa) protein detected with anti-Transferrin receptor 2 (Antibodies; 1:1000) antibody.

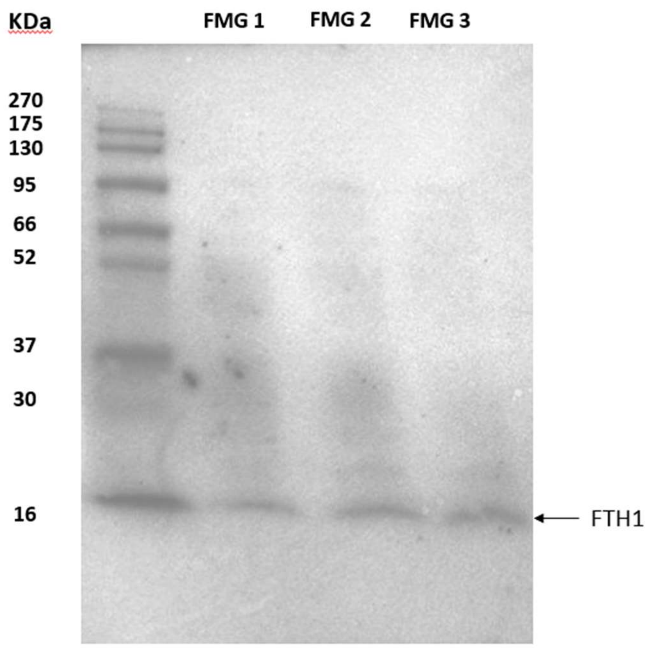

Figure S3. Western blot membrane of FTH1 (~20 kDa) protein detected with anti-Ferritin heavy chain 1 (Antibodies; 1:500) antibody.

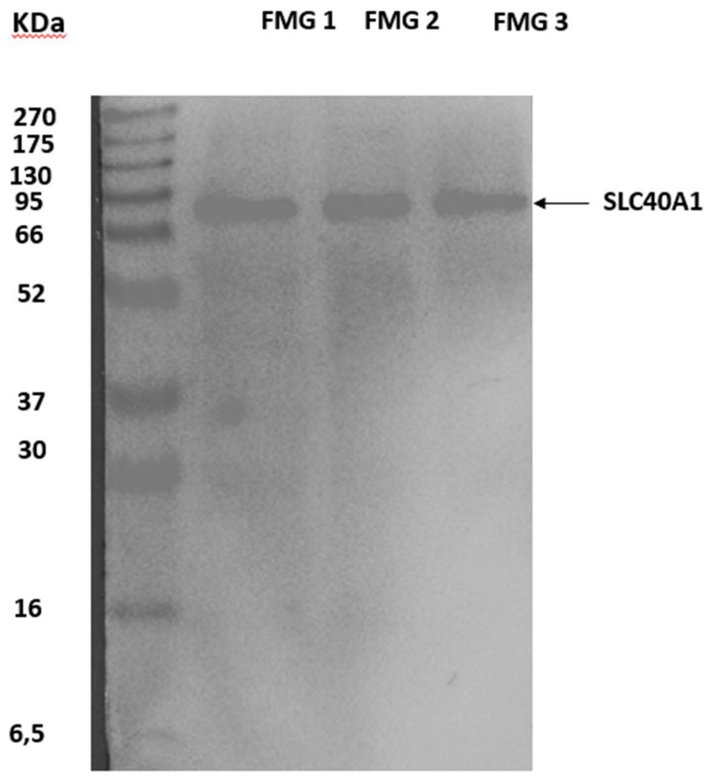

Figure S4. Western blot membrane of SLC40A1 (~62 kDa) protein detected with anti-SLC40A1 (Elabscience; 1:1000) antibody.

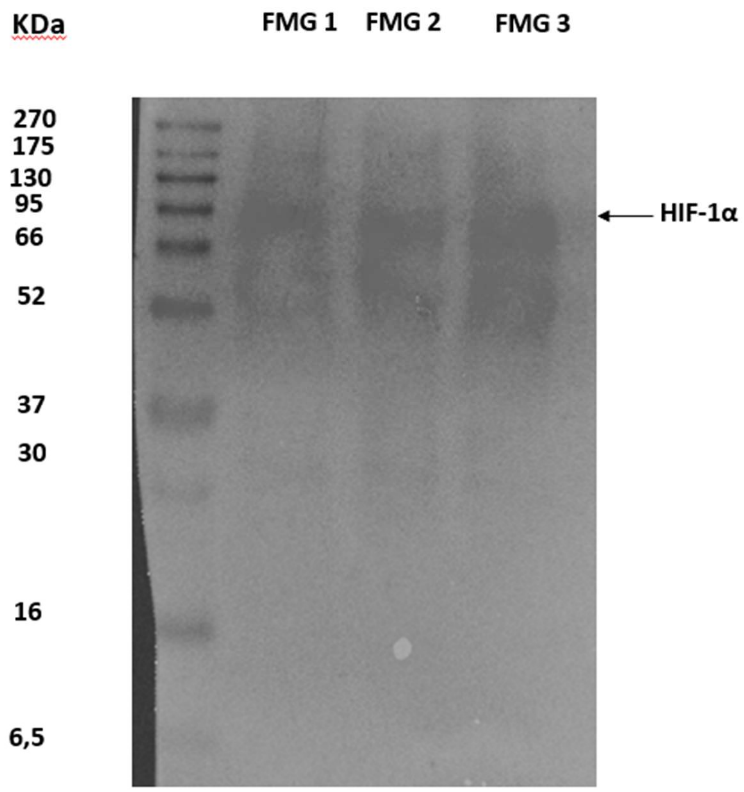

Figure S5. Western blot membrane of HIF-1α (~93 kDa) protein detected with anti-HIF-1α (Abcam, 1:2000, Cambridge, UK) antibody.

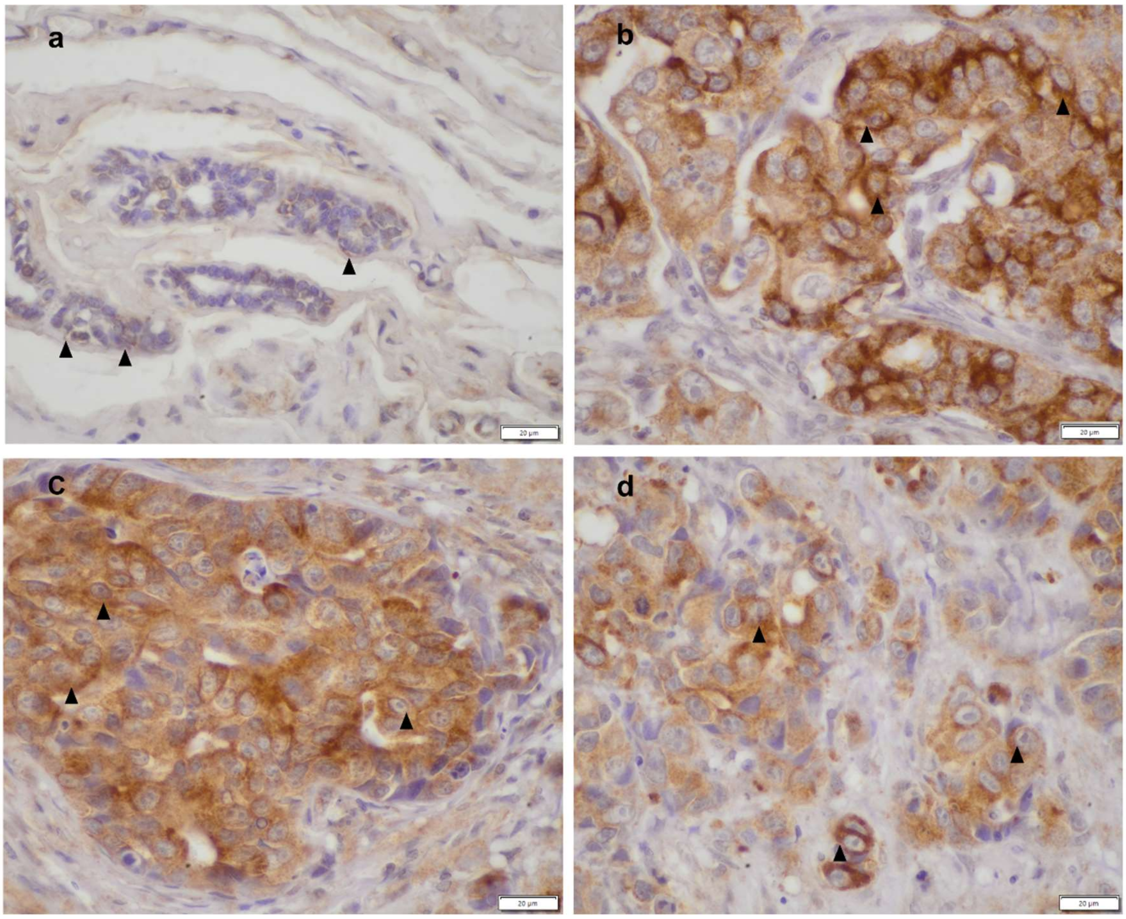

Figure S6. Feline mammary tissue. TfR1 immunostaining. (a) Normal mammary gland showing scattered membranous labeling in epithelial cells (arrowheads); (b) non-metastatic mammary carcinoma showing strong cytoplasmic labeling in tumor cells (arrowheads); (c) metastatic mammary carcinoma showing strong cytoplasmic labeling in tumor cells (arrowheads) and necrosis; (d) tributary lymph node with metastatic cells showing cytoplasmic labeling (arrowheads). **60×, scale bar = 20 µm.**

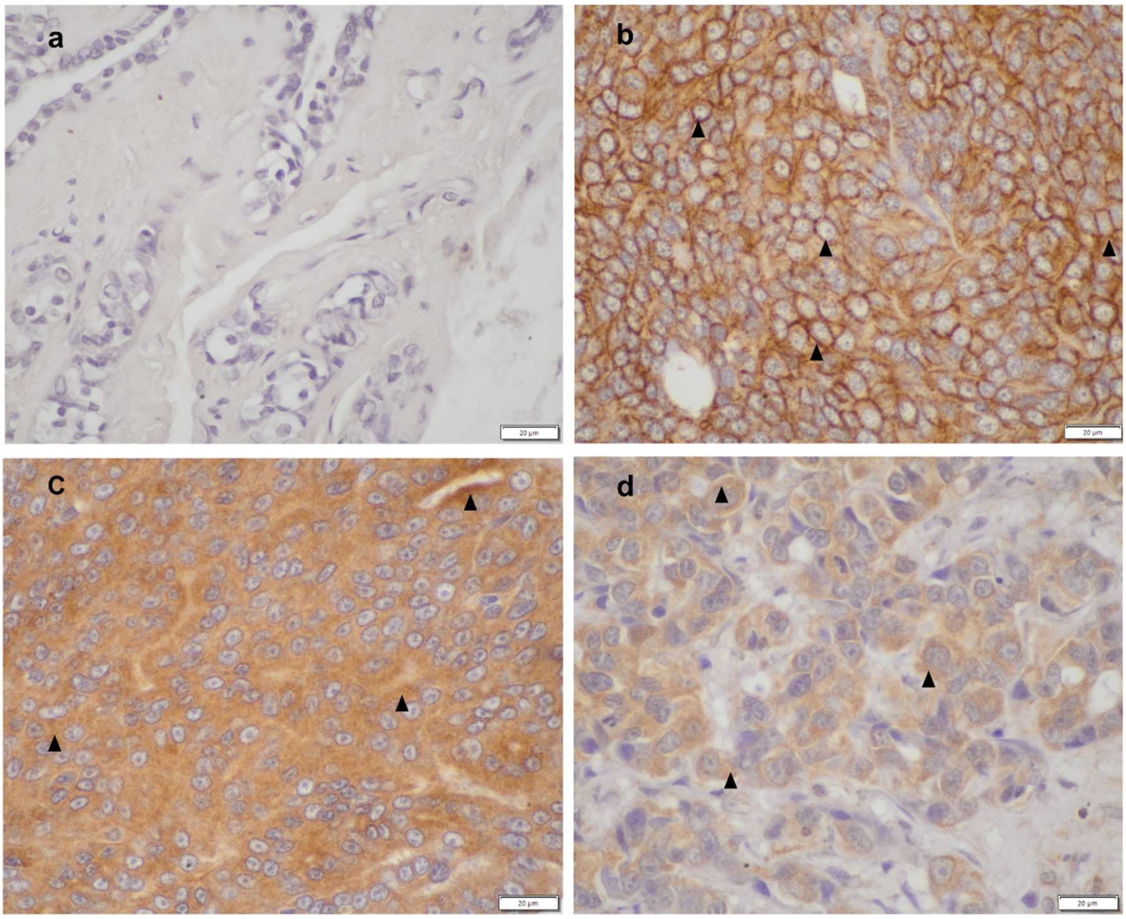

Figure S7. Feline mammary tissue. TfR2 immunostaining. (a) Normal mammary gland showing few labeled epithelial cells; (b) non-metastatic mammary carcinoma showing strong membranous labeling in tumor cells (arrowheads); (c) metastatic mammary carcinoma showing very strong cytoplasmic labeling in tumor cells (arrowheads); (d) tributary lymph node with metastatic cells showing cytoplasmic labeling in tumor cells (arrowheads). **60×, scale bar = 20 µm.**

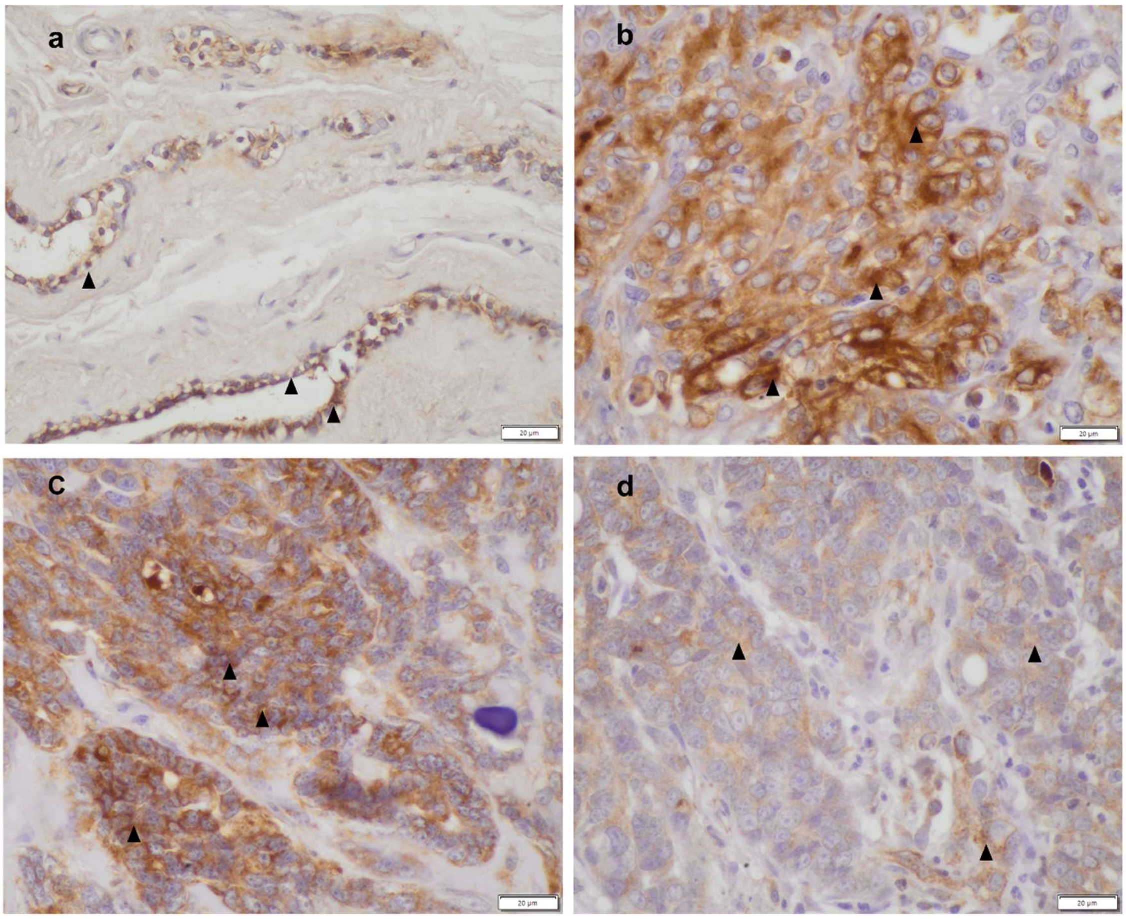

Figure S8. Feline mammary tissue. FTH1 immunostaining. (a) Normal mammary gland showing few labeled epithelial cells (arrowheads); (b) non-metastatic mammary carcinoma showing strong labeling in tumor cells (arrowheads); (c) metastatic mammary carcinoma showing strong cytoplasmic labeling in tumor cells (arrowheads); (d) tributary lymph node with metastatic cells showing cytoplasmic labeling in tumor cells (arrowheads). **60×, scale bar = 20 µm.**

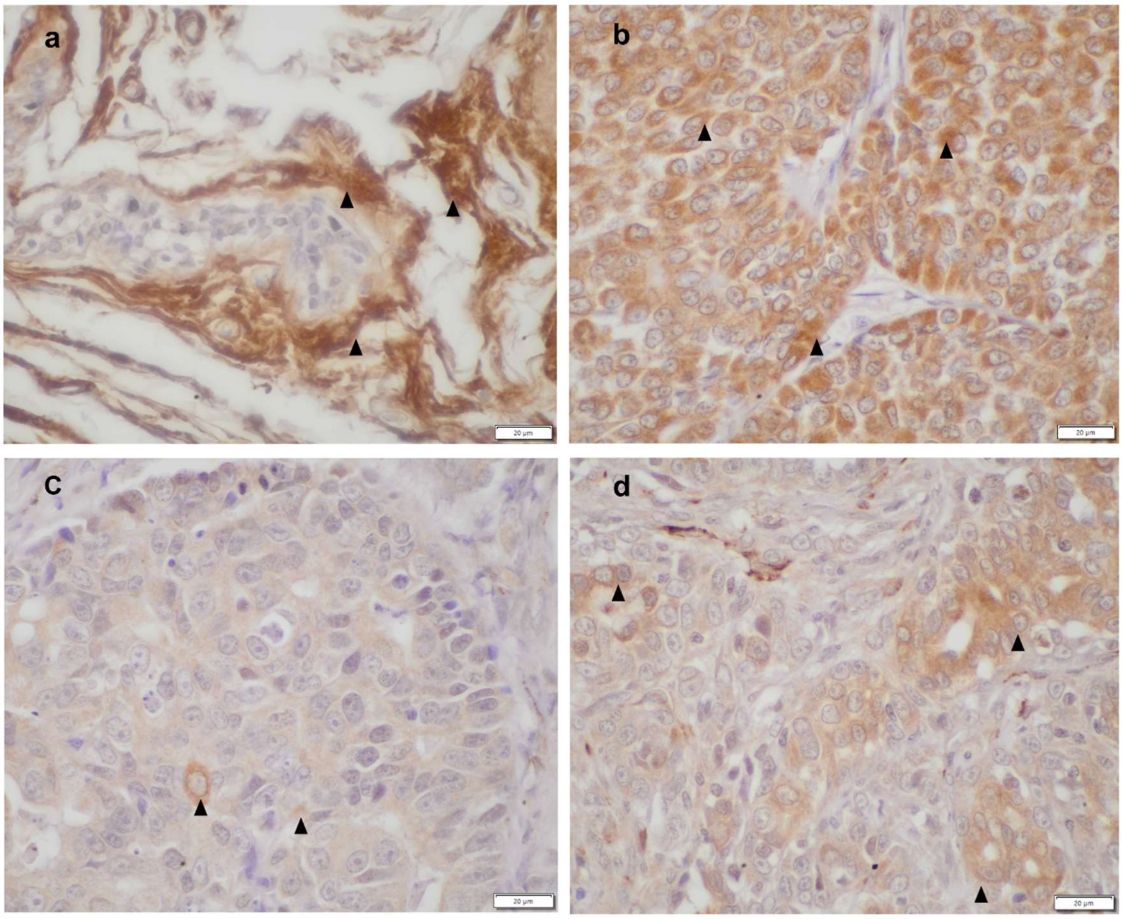

Figure S9. Feline mammary tissue. SLC40A1 immunostaining. (a) Normal mammary gland showing no labeled epithelial cells; (b) non-metastatic mammary carcinoma showing strong cytoplasmic labeling in tumor cells (arrowheads); (c) metastatic mammary carcinoma showing weak cytoplasmic labeling in a few tumor cells (arrowheads); (d) tributary lymph node with metastatic cells showing cytoplasmic labeling in tumor cells (arrowheads). **60×, scale bar = 20 µm.**

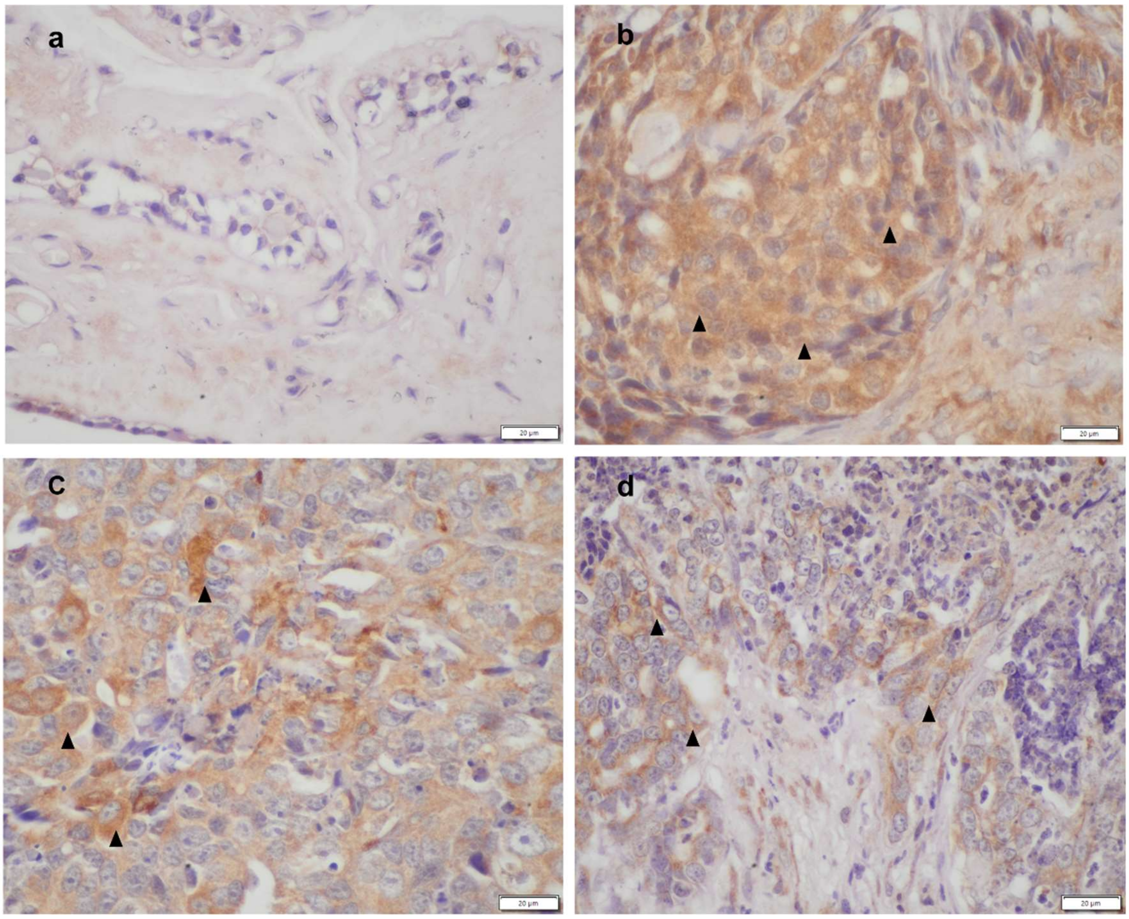

ù

Figure S10. Feline mammary tissue. HIF-1α immunostaining. (a) Normal mammary gland showing no labeled cells; (b) non-metastatic mammary carcinoma showing strong cytoplasmic labeling in tumor cells (arrowheads); (c) metastatic mammary carcinoma showing cytoplasmic labeling in a few tumor cells (arrowheads); (d) tributary lymph node with metastatic cells showing moderate cytoplasmic labeling in tumor cells (arrowheads). **60×, scale bar = 20 µm.**
